# Polymer Chemical Identity as a Key Factor in Microplastic–Insecticide Antagonistic Effects during Embryogenesis of Sea Urchin *Arbacia lixula*

**DOI:** 10.3390/ijms24044136

**Published:** 2023-02-18

**Authors:** Petra Burić, Ines Kovačić, Lara Jurković, Serkan Tez, Rahime Oral, Nediljko Landeka, Daniel M. Lyons

**Affiliations:** 1 Faculty of Natural Sciences, Juraj Dobrila University of Pula, 52100 Pula, Croatia; 2 Faculty of Educational Sciences, Juraj Dobrila University of Pula, 52100 Pula, Croatia; 3 Center for Marine Research, Ruđer Bošković Institute, 52210 Rovinj, Croatia; 4 Faculty of Fisheries, Ege University, Bornova, 35100 Izmir, Turkey; 5 Teaching Institute of Public Health of the Istrian County, 52100 Pula, Croatia

**Keywords:** coastal, cypermethrin, embryotoxicity, leachate, pesticide, PMMA, polymethylmethacrylate, polystyrene, pyrethroid, urban wastewater

## Abstract

As a proxy for pollutants that may be simultaneously present in urban wastewater streams, the effects of two microplastics—polystyrene (PS; 10, 80 and 230 μm diameter) and polymethylmethacrylate (PMMA; 10 and 50 μm diameter)—on fertilisation and embryogenesis in the sea urchin *Arbacia lixula* with co-exposure to the pyrethroid insecticide cypermethrin were investigated. Synergistic or additive effects were not seen for plastic microparticles (50 mg L^−1^) in combination with cypermethrin (10 and 1000 μg L^−1^) based on evaluation of skeletal abnormalities or arrested development and death of significant numbers of larvae during the embryotoxicity assay. This behaviour was also apparent for male gametes pretreated with PS and PMMA microplastics and cypermethrin, where a reduction in sperm fertilisation ability was not evidenced. However, a modest reduction in the quality of the offspring was noted, suggesting that there may be some transmissible damage to the zygotes. PMMA microparticles were more readily taken up than PS microparticles, which could suggest surface chemical identity as potentially modulating the affinity of larvae for specific plastics. In contrast, significantly reduced toxicity was noted for the combination of PMMA microparticles and cypermethrin (100 μg L^−1^), and may be related to less ready desorption of the pyrethroid than PS, as well as cypermethrin activating mechanisms that result in reduced feeding and hence decreased ingestion of microparticles.

## 1. Introduction

There has been a dramatic increase in interest in plastics, and in particular microplastics, in the environment over the last decade. This is related to growing awareness of the ever-increasing quantities of plastics reaching various environmental compartments, especially aquatic systems, where up to 5 trillion pieces of plastic have been estimated to be present, and their potentially harmful effects on living organisms [1,2,3,4,5]. Further, plastics’ long residence times due to resistance to rapid degradation only enhance the potential risk to biota. Such risk extends to food webs, where plastics, as they are gradually broken down into smaller pieces and particles due to aging by UV light, mechanical stresses and microbes [6], may be transferred among trophic levels and reach as far as humans [7]. Investigations have shown that even sea salt used in kitchens is contaminated with plastic particles, thus representing a more direct pathway to ingestion by humans [8]. In addition, cosmetics and personal hygiene products such as toothpaste also contain plastic microbeads and represent another source of plastics in the human body [9]. Indeed, even from their earliest days, humans are exposed to microplastics, as it has been shown that infants may potentially ingest large quantities of polypropylene microparticles of 0.015–4.550 × 10^6^ day^−1^, deriving from plastic feeding bottles [10]. Thus, the growing presence of plastic microparticles around us and increasing concern that these may represent a toxicity risk, have given rise to a broad range of investigations in a wide spectrum of organisms [11].

However, microplastics are not a homogeneous class, but rather a complex range of materials, as not only are the physical size and specific polymer important in defining their behaviour and impact on the environment but also their chemical composition. This specifically refers to chemicals that may have been used in their production or added afterwards to impart particular properties to the materials, and encompass such compounds as plasticisers and dyes. Added to this is the fact that plastics also contain monomers and oligomers deriving from production processes due to incomplete polymerisation. The potential for leaching of monomers, short-chain polymers and a range of chemical additives demonstrates how the chemical identity of plastics in the environment is an extremely complex issue that is just beginning to be addressed [12,13].

Aquatic organisms have received the most attention to date as models for toxicity testing [5]. Of invertebrates, mussels have been one of the mainstay models due to the prodigious quantity of water filtered daily and their potential to accumulate pollutants in the water column. Mussels have been shown to ingest, for example, polyvinylchloride (PVC) microparticles, although no significant toxicity was reported after chronic exposure [14]. However, the leachates of polystyrene and PVC microparticles were found to cause significant modulation of catalase and glutathione peroxidase activities, dopamine levels in mussels, and embryonal development [15,16]. Ingestion by a range of other organisms including dietary exposure of gilt-head seabream *Sparus aurata* to six different microplastics including PVC, PS and polyethylene (PE) did not result in accumulation of microplastics or induction of stress [17], and crustacean *Aristeus antennatus* or *Daphnia magna* [18,19] did not show significant deleterious effects from microplastics either. Similar to embryonal development in mussels, there was a corresponding lack of adverse effects on sea urchin *Paracentrotus lividus* larvae after exposure to PE [20]. On the contrary, PVC, PE, PS, polyamide (PA) and polypropylene (PP) microplastics induced intestinal damage and oxidative stress in *Caenorhabditis elegans* and growth inhibition in *Danio rerio* [21], and mild developmental delays in *Sphaerechinus granularis* and *P. lividus* larvae after exposure to PS and polymethylmethacrylate (PMMA) microparticles [22,23]. Thus, a clear consensus has yet to form on whether microplastics may present a significant risk to biota, particularly in terms of potentially different effects deriving from variously sized particles or species-dependent toxicity [24,25,26].

Irrespective of cases where microplastics alone may represent a risk to biota, microplastics are not present in the environment in isolation, but in many cases may be present concomitantly with other anthropogenic materials such as engineered nanoparticles, persistent organic pollutants, pharmaceuticals and pesticides [27]. The widespread use of pesticides in agriculture increases the likelihood of them making their way from one environmental compartment to another, such as from groundwater to streams and potentially to estuaries and coastal waters [28]. Insecticides commonly used in urban areas, such as those used for citywide control of mosquito populations, may also find their way to wastewater streams, which potentially may encounter microplastics. It is the interplay between such materials, for example, the adsorption of hydrophobic chemicals on microplastics [20], that may modulate residence times and potentially give rise to additive or synergistic toxicity effects in biota, hence representing a significant cause for concern.

While a range of legacy organic pollutants such as PAHs sorbed on microplastics have been investigated for toxicity, there are few data available on potentially synergistic or additive effects of pyrethroids and microplastics concurrently present in the aquatic environment on biota such as sea urchins. Indeed, the sea urchin in particular has proven a particularly valuable model, as its early life development phases and the functioning of its immune system are analogous to corresponding processes in humans. Thus, as a proxy for these pollutants that may be simultaneously present in urban wastewater streams and eventually reach coastal waters, the present study examines the effects of the pyrethroid neurotoxin cypermethrin on fertilisation, embryogenesis and transmissible damage to offspring in the sea urchin *Arbacia lixula* in the presence of different sizes of PS and PMMA microplastics.

## 2. Results

### 2.1. Adsorption Analysis

**Figure 1:**
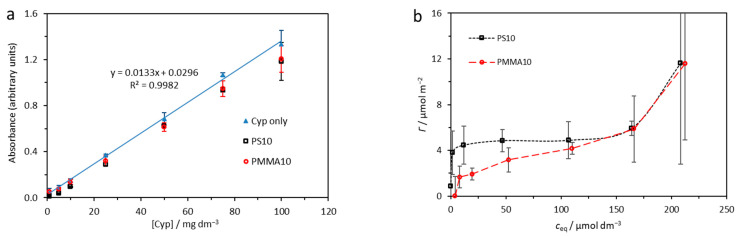
(**a**) Absorbance (mean ± SD) at a wavelength of 279 nm as a function of cypermethrin (γ_0_(Cyp) = [Cyp]) added to 10 μm polystyrene (PS10) and polymethylmethacrylate (PMMA10) microparticle suspensions. (**b**) Surface concentration (mean ± SD) of cypermethrin (*Γ*) on microplastics with respect to cypermethrin concentration (c_eq_) in solution.

Adsorption data were obtained as mean values of three individual experiments at a wavelength of 279 nm and shown in Figure 1. Good linearity was noted for absorbance of cypermethrin over a wide range of concentrations at the selected wavelength (R^2^ = 0.9982), while absorbance measurably decreased after addition of PS and PMMA microparticles, indicating removal of cypermethrin from solution (Figure 1a).

**Figure 2:**
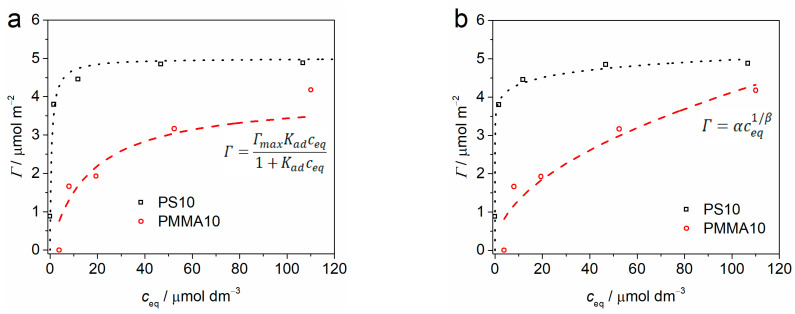
(**a**) Langmuir and (**b**) Freundlich adsorption isotherms for cypermethrin on 10 μm polystyrene (PS10) and polymethylmethacrylate (PMMA10) microparticles.

The change in concentration of cypermethrin on the microplastic surface with change in concentration of cypermethrin in solution is shown in Figure 1b. The virgin microplastics show an initial rapid uptake of cypermethrin that then slows, even as the concentration of cypermethrin in solution continues to increase, while at high cypermethrin concentrations the surface adsorption again begins to rise. This behaviour indicates initial rapid formation of a monolayer of cypermethrin on the microplastics’ surfaces followed by growth of a cypermethrin multilayer at high cypermethrin concentrations. The Langmuir and Freundlich adsorption isotherms were calculated for monolayer adsorption of cypermethrin on PS10 and PMMA10, and are shown in Figure 2. The fit obtained by the Langmuir model (Figure 2a) was similar to the very good fit obtained by the empirical Freundlich model (Figure 2b), indicating that the assumptions of the Langmuir model may not be wholly unsuitable for understanding the nature of the adsorption of cypermethrin on PS and PMMA. Further, it may be noted that the adsorption constant K_ad_ is far greater for PS10 than for PMMA10, and sorptive capacity of the PS10 surface is greater than PMMA10 (Table 1), indicating more rapid and greater uptake of cypermethrin by PS10.

### 2.2. Embryotoxicity Assay

**Figure 3:**
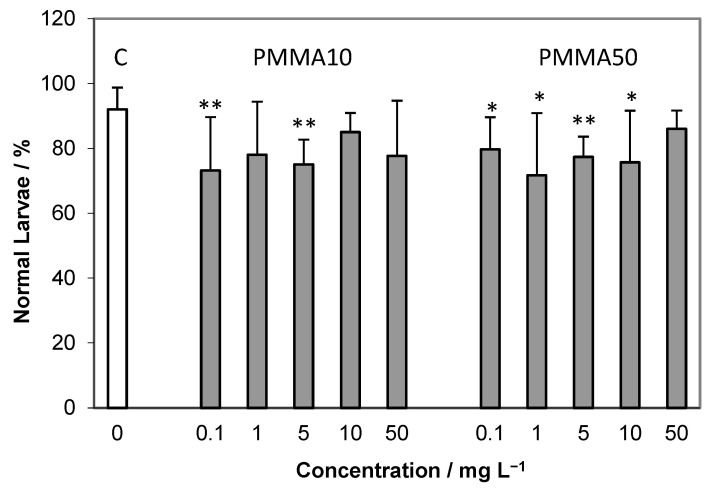
Percentage (mean ± SD) of normally developed plutei larvae after 72 h exposure to various concentrations of 10 and 50 μm PMMA microparticles (C—control). * *p* < 0.05, ** *p* < 0.01.

Scoring of embryos of *A. lixula* at 72 h post-fertilisation showed both normal larvae and a range of adverse effects including arrested development, skeletal malformations and dead larvae. The impact of PMMA particles only on developing embryos is given in Figure 3. While the control showed about 90% normal larvae after three days, all treatments with PMMA particles reduced the percentage of normal larvae to about 70–80%, with a concomitant significant increase in larvae showing retarded development and developmental defects. No concentration-dependent decrease in normal larvae was noted for either the 10 μm or 50 μm microparticles.

**Figure 4:**
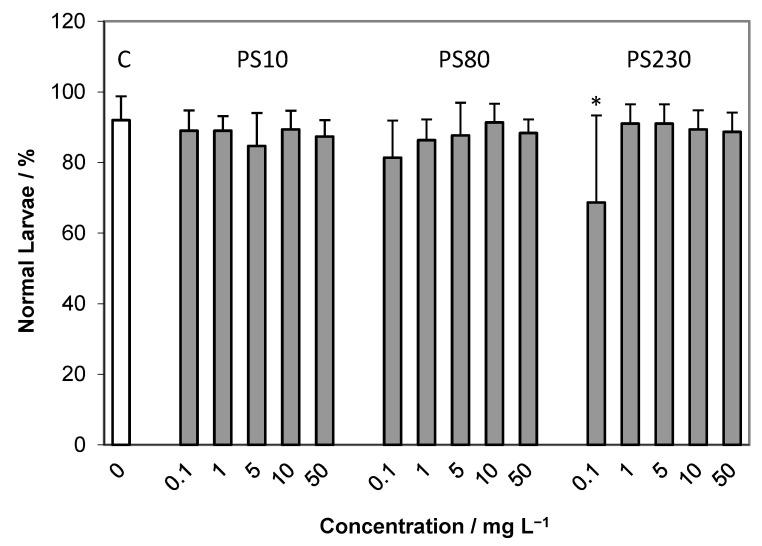
Percentage (mean ± SD) of normally developed plutei larvae after 72 h exposure to various concentrations of 10, 80 and 230 μm PS microparticles (C—control). * *p* < 0.05.

Exposure of developing embryos to PS microparticles with diameters of 10, 80 and 230 μm induced only a slight reduction in normal plutei with respect to control samples, to about 80–85%, with no clear trend evidenced with increasing microparticle concentration (Figure 4).

**Figure 5:**
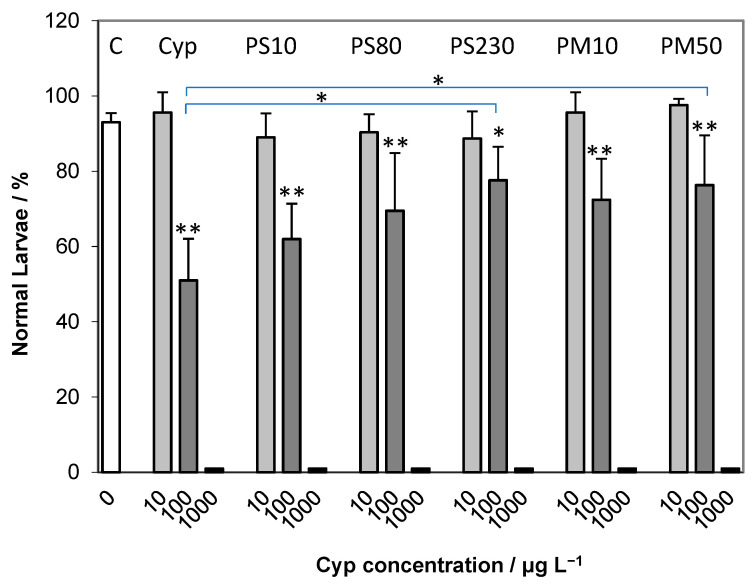
Percentage (mean ± SD) of normally developed plutei larvae after 72 h co-exposure to 50 mg L^−1^ microparticles and various concentrations of cypermethrin (C—control, Cyp—cypermethrin only). Significant differences compared to the control (** *p* < 0.01) are shown at the top of the columns, while significant differences compared to cypermethrin only (* *p* < 0.05) are denoted by the horizontal bars.

Cypermethrin did not result in a reduction in normally developed plutei at a concentration of 10 μg L^−1^, while at the higher concentration of 100 μg L^−1^ a significant difference (*p* < 0.01) from the control was noted, where only about 50% of the larvae were normally developed and the remainder showing developmental delay (Figure 5). Upon addition of the combination of PS or PMMA microparticles (50 mg L^−1^) with cypermethrin (10 μg L^−1^) to the developing zygotes, no decrease in normally developed plutei was noted after 72 h, with values close to the control and to treatments with cypermethrin only, indicating a lack of additive or synergistic effects at these concentrations. In contrast, the combination of the polymer microparticles and cypermethrin at the higher concentration of 100 μg L^−1^ showed significant differences (*p* < 0.01) to the control in terms of reduced percentage of normal plutei for all microparticles. However, an increase in percentage of normal plutei compared to cypermethrin alone was noted in the presence of all microparticles, with the largest diameter PS (230 μm) and PMMA (50 μm) showing significant (*p* < 0.05) increases in normally developed plutei. At the highest cypermethrin concentration of 1000 μg L^−1^, with or without microplastics, all larvae were severely developmentally delayed or dead.

### 2.3. Spermiotoxicity Assay

A second series of experiments was conducted to probe any effects the PS and PMMA microplastics or cypermethrin may have on the fertilisation ability of sperm. After pretreatment with toxicants, the sperm in nearly all cases showed the same ability to fertilise eggs with no significant differences to the controls (Supplementary Information: Figures S1 and S2), hence indicating no adverse effect on fertilisation rate. However, the quality of the offspring deriving from the sperm that had been pretreated with microplastics or cypermethrin showed greater effects than in earlier experiments where developing embryos were first exposed to toxicants only after fertilisation had occurred. Compared to the 90% normal plutei of the control, offspring of sperm-treated samples showed a reduction in normal larvae to about 65–85%, with the greatest effect noted for PS230, where about 70% normal plutei were consistently observed at particle concentrations ≥5 mg L^−1^ (SI: Figure S3). Clear trends in percentage of normally developed plutei were not apparent, although some significant differences from controls confirmed the negative impact of the microparticles in the tested concentration range. Larger PMMA microparticles also gave a lower percentage of normal larvae, with the 50 μm PMMA-treated samples typically giving about 70% normal plutei, irrespective of concentration (SI: Figure S4). Statistically significant differences from controls were noted for low concentrations of both PMMA10 and PMMA50. Interestingly, cypermethrin-treated sperm did not give a very large decrease in normal offspring, even at the highest concentrations (100 μg L^−1^), with about 80% normal larvae noted (SI: Figure S4).

Furthermore, to determine if leachate from the microplastics may cause a negative effect on embryonal development, filtered seawater in which the microplastics had been aged for 1 month was used to pretreat the sperm for 1 h. Fertilisation success of these sperm was above 95%, and no significant difference in the quality of the offspring 72 h post-fertilisation compared to untreated controls was noted (SI: Figure S5).

**Figure 6:**
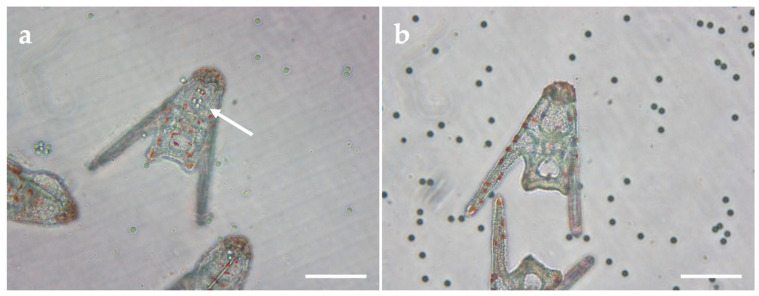
Representative images of *A. lixula* embryos with (**a**) ingested 10 μm PMMA microparticles (indicated by arrow) and (**b**) lack of ingestion after co-exposure to microparticles and cypermethrin (scale bar 100 μm).

In developing larvae, microplastics with diameters of 50–230 μm were not evidenced in the gut of plutei. However, microplastics of 10 μm diameter were often found to be ingested, with a representative image showing their presence in the gut given in Figure 6a. PMMA microparticles were found to be ingested far more commonly and in greater quantity than their PS analogues. However, when embryos were co-exposed to 10 μm microplastics and insecticide, uptake of microplastics was not observed (Figure 6b).

## 3. Discussion

The sea urchin has proven a valuable model in toxicity testing due to its ease of rearing under laboratory conditions, well-defined morphological characters and physiology, and extensive data in the literature on its behaviour when exposed to a broad range of pollutants [29,30]. In the present study, the sea urchin *A. lixula* was used as a test model for evaluating the potential for co-exposure to materials, which may be simultaneously present in urban wastewater streams and may subsequently be transported to other aquatic systems, to cause adverse effects during embryogenesis. In all cases, there is generally little evidence for strong toxicity due to the action of both toxicants at the same time. Synergistic or additive effects were not seen for plastic microparticles in combination with synthetic pyrethroid cypermethrin based on evaluation of skeletal abnormalities or developmental delay and death of significant numbers of larvae during the embryotoxicity assay. This behaviour was also apparent for sperm pretreated with PS and PMMA microplastics and cypermethrin, where a reduction in sperm fertilisation ability was not evidenced. However, a modest reduction in the quality of the offspring was noted, suggesting that there may be some transmissible damage to the zygotes. While there was some transfer of toxicant (i.e., with sperm) to the egg suspension after the sperm pretreatment, the toxicant was diluted such that its final concentration was 200× less that the corresponding treatment in the embryo development test. Thus, it is generally unlikely that the toxicant is responsible for this effect during embryonic development of the zygotes. However, in other systems it should be noted that toxicity has been found for extremely low concentrations of endocrine-disrupting chemicals (EDC) [31]. Therefore, it is possible that low concentrations of cypermethrin or potentially leachates from the microplastics may give rise to EDC-like effects that may manifest in developmental arrest or deformities in urchin embryos [32]. Indeed, abnormalities in the embryonic development of *P. lividus* zygotes after being treated with leachates from PS and high-density polyethylene fragments were noted [33], with similar data being reported for PS microbead-induced developmental defects [34,35]. Leachates from PE fragments were also found to negatively impact larval development of *Lytechinus variegatus*, with lower numbers of normal larvae noted after exposure [36]. A similar effect was noted for leachates from PVC microparticles, where a developmental arrest was observed after fertilisation, with morphological changes in some embryos that survived [37]. Further circumstantial evidence that EDC-like behaviour may be caused by leachates derives from studies of PP fragments where leachates resulted in a dose-dependent increase in deformities in the brown mussel *Perna perna*, with the lowest concentrations showing significant effects [15]. Evaluation of the potential for microplastic leachate to cause negative effects on the fertilisation ability of the sperm or on the quality of the subsequent offspring was also carried out in the present work. No evidence was found that leachates from the PS or PMMA microplastics had any deleterious effects, although this may be related to the fact that the particles were virgin microplastics that had not aged in the real environment.

It is in real environmental matrices that breakdown processes, typically based on UV light and mechanical degradation, coupled with the potential to adsorb a wide range of toxicants including heavy metals, pharmaceuticals, persistent organics and hydrocarbons, may significantly enhance the toxicity of microplastics compared to their virgin analogues [38,39]. Interestingly, the co-exposure of developing urchin embryos to a commercial formulation of the pyrethroid cypermethrin and microplastics showed reduced toxicity of the insecticide to the urchin larvae compared to toxicity of the cypermethrin in the absence of microplastics. This antagonistic effect may be related to the adsorption of cypermethrin on the surface of the polymer microparticles that reduces its ability to interact with the larvae. Data presented herein on the absorption behaviour of cypermethrin on PS and PMMA microparticles indicate the ability of the microparticles to sequester low concentrations of the insecticide. A good fit was observed for the Freundlich adsorption isotherm, suggesting some heterogeneity in adsorption site on the particle surface and a dynamic exchange process, and correlates with reports on the sorption of polycyclic aromatic hydrocarbons on polystyrene nanoparticles [40]. Such ready adsorption likely derives from a number of mechanisms, including hydrophobicity-driven interactions between aromatic molecule and particle surface [41], hydrogen bonding and, for example, π–π interactions between the aromatic rings of the insecticide and PS [42]. A comparison of adsorption of α-cypermethrin on PS and PMMA microparticles showed similar behaviour where 41 μm PS more readily sorbed the organic molecule than 48 μm PMMA over 48 h due to the lesser polarity of the former and likely ability of the α-cypermethrin benzene rings to more easily intercalate between the polymer chains of PS [43]. However, in the present work, at higher concentrations more than one layer of cypermethrin may form on the microplastics and the β value indicates that significantly more molecules (eightfold) can desorb from the PS surface compared to that of the PMMA microparticles. Thus, should there be ingestion of microplastics with surface-adsorbed cypermethrin, the pyrethroid may more readily dissociate from the microplastic inside the organism in the case of PS microparticles resulting in more pronounced toxicity. This is in line with the observation that, while the microparticles reduced the overall toxicity of cypermethrin, co-exposure to cypermethrin and PMMA resulted in less developmental delay compared to the corresponding cypermethrin-PS co-exposure. In contrast, a recent report on the development of a pyrolysis–GC/MS-based analytical technique for estimating adsorption/desorption of organic molecules on a range of nano- and microplastics noted that α-cypermethrin showed greater sorption on 48 μm PMMA than 40 and 41 μm PS microparticles [44]. However, this may be related to the relatively short contact time in which sorption processes had not yet reached equilibrium, and PS over a longer period would be expected to adsorb a greater quantity of α-cypermethrin than PMMA.

The uptake of microplastics by the plutei also showed interesting differences between polymers. PMMA microparticles when introduced in combination with cypermethrin were not evidenced in the gut, while exposure to PMMA microparticles alone often resulted in uptake with microparticles clearly seen in the gut. It is therefore interesting to note that PMMA microparticles were more readily taken up than PS microparticles, which may suggest surface chemical identity, including polarity, as potentially having some influence in modulating the affinity of larvae for specific plastics. However, why larvae reject ingestion of PMMA microparticles in the presence of cypermethrin remains a topic of speculation, although it may be that the presence of the insecticide activates various mechanisms, including, for example, disruption of cellular sodium and calcium channels, which eventually may be reflected in reduced feeding and hence decreased ingestion of microparticles.

## 4. Materials and Methods

### 4.1. Materials

Polymethylmethacrylate and polystyrene virgin microparticles with nominal diameters of 10 and 50 μm (PMMA) and 10, 80 and 230 μm (PS) were obtained from Microbeads SA, Skedsmokorset, Norway and used without modification. Aquacyp insecticide (100 g L^−1^ cypermethrin as the active component (M_w,Cyp_ = 416.3 g mol^−1^) was obtained as an over-the-counter commercial product (Colkim, Bologna, Italy), while chromic(III) potassium sulphate dodecahydrate was purchased from Carl Roth, Karlsruhe, Germany.

### 4.2. Adsorption of Cypermethrin on Plastic Microparticles

To solutions of cypermethrin, with concentrations in the range 1–100 mg L^−1^ (2.4–240 μmol L^−1^), PS and PMMA microparticles of 10 μm diameter were added such that the final microplastic concentration was 5 g L^−1^. The suspensions were gently mixed for 30 min at 25 °C, upon which the microplastics were separated by centrifugation at 1800× *g* for 1 min. The concentration of cypermethrin in supernatant solution was determined spectrophotometrically on a Shimadzu UV-1800 spectrophotometer (Kyoto, Japan), with absorbance measured in 10 mm path length quartz cuvettes at a wavelength of 279 nm, which is a characteristic band for protonated benzene rings [45].

The surface concentration (*Γ*) of cypermethrin on microplastics was calculated by determining the difference between cypermethrin (c_0_) added to the microparticle suspension and cypermethrin (c_eq_) remaining in solution after 30 min ((c_0_ − c_eq_) V) divided by the surface area of microplastic.

The surface area (*S*) was calculated by:

$$S=\frac{6W}{PD}m^{2}$$

where *W* is the mass of the polymer in grams, *P* is the density of the polymer (PS = 1.05, PMMA = 1.20) and *D* is the diameter of particles in micrometres (10 μm). The surface areas of PS and PMMA were thus calculated to be 0.0120 m^2^ and 0.0137 m^2^, respectively.

### 4.3. Sea Urchin Embryo Development Test

Sea urchins (*Arbacia lixula*) were collected in February 2019 at a depth of 1–2 m off the coast of Izmir, Turkey and gametes were harvested, eggs fertilised and embryos reared as reported previously [25]. Briefly, the gonads were removed and those from three females placed in filtered seawater (FSW) while those from two males were held ‘dry’ in separate containers. Eggs from each female, at a concentration of ~1000–2000 mL^−1^, were fertilised by sperm from each male at a final dilution of ~100,000× (giving 6 permutations), and 1 h post fertilisation 1 mL of these 6 suspensions was placed in the respective wells of 6-well polystyrene multi-well tissue culture plates to which had previously been added 9 mL FSW and the toxicant to be tested. Control samples comprised of embryos reared in the absence of toxicants. Embryos were reared in the presence of only PMMA or PS microparticles at concentrations of 0.1, 1, 5, 10 and 50 mg L^−1^ or in the presence of cypermethrin (10–1000 μg L^−1^) adsorbed on microplastics. Embryo exposure lasted until the pluteus larval stage 72 h post-fertilisation and experiments were carried out in at least quintuplicate. At the end of the experiment 100 μL of 10^−2^ M chromic(III) potassium sulphate dodecahydrate was added to each well and after 10 min the embryos were scored for developmental defects. In each well, 100 plutei were scored for the percentage of normal larvae, developmentally delayed larvae (less than half normal size), malformed larvae and the presence of skeletal defects, embryos/larvae unable to achieve the pluteus stage and dead embryos or larvae.

### 4.4. Spermiotoxicity Test and Offspring Quality

Sperm diluted 100× in FSW were exposed to the individual microplastics (various polymers and sizes) or to cypermethrin for 10 min. upon which a 50 μL aliquot was withdrawn and added to 10 mL of egg suspension. Fertilisation success was noted as the percent of fertilised eggs (as live cleaving embryos) 1–3 h post-fertilisation. These embryos were allowed to develop for 72 h post-fertilisation and were scored for developmental defects, as described above.

### 4.5. Statistical Analysis

Results are presented as means ± standard deviation of at least 5 replicates. Data were examined for normality (Shapiro–Wilk test) and homogeneity of variance (Levene’s test). Those that satisfied these requirements were tested by one-way ANOVA followed by post hoc Tukey’s test. For data that did not satisfy the assumptions required for parametric testing, a nonparametric approach was taken, analysing variance among data by the Kruskal–Wallis test and Mann–Whitney U test. The *p* < 0.05 level was selected to indicate when differences from control samples were significant.

## 5. Conclusions

Sea urchin embryos exposed to the insecticide cypermethrin showed less negative outcomes in terms of number of abnormally developed plutei, arrested development and dead larvae when PS and PMMA polymer microparticles were present during embryo exposure to the pyrethroid. Similarly, for male gametes exposed to cypermethrin in the presence of PS and PMMA, there was no reduction in fertilisation ability, although some transmissible damage to offspring was noted. In plutei that had started to actively feed by the end of the exposure period, 10 μm PMMA microparticles was observed in the gut while 10 μm PS microparticles were not seen to be taken up, suggesting surface chemical identity may be a factor in particle uptake. Though PMMA was internalised, it had adsorbed less cypermethrin than PS and may have held that quantity of cypermethrin more tightly, leading to an overall antagonistic effect between the PMMA microparticles and cypermethrin in terms of toxicity to embryos. In contrast, higher toxicity for the combination of PS microparticles and cypermethrin may be related to initial greater pesticide uptake and fast desorption kinetics (possibly after some microparticle uptake into the gut) which could have quickly affected feeding patterns of the plutei, eventually resulting in less PS microparticle uptake overall.

Ultimately, while there is an increasing number of studies on co-exposure to pollutants and microplastics focusing on hydrophobic organic molecules, for example, the combined effect of PE microparticles and benzophenone [46] or 4-*n*-nonylphenol [20] on *P. lividus*, these and the data reported in the present work indicate a need to extend studies to a wider range of pollutants and microplastics so as to draw broader conclusions about the mode of action of such combinations, and if microplastics should continue to be considered materials of concern, either on their own or in association with other pollutants, particularly in light of demonstrated antagonistic effects.

## Figures and Tables

**Table 1:** Langmuir (maximal surface concentration *Γ*_max_, adsorption constant K_ad_) and Freundlich (α, β) adsorption isotherm parameters for microplastic-adsorbed cypermethrin.

	*Γ*_max_/μmol m^−2^	K_ad_/μmol^−1^ dm^3^	α	β
PS10	5.0 ± 0.3	1.53 ± 0.98	3.8 ± 0.1	16.5 ± 2.2
PMMA10	4.0 ± 0.9	0.06 ± 0.04	0.4 ± 0.2	2.0 ± 0.5

## Data Availability

Not applicable.

## Supplementary Materials

The following supporting information can be downloaded at https://www.mdpi.com/article/10.3390/ijms24044136/s1.
