# Supplementary material for: Polymer Chemical Identity as a Key Factor in Microplastic–Insecticide Antagonistic Effects during Embryogenesis of Sea Urchin Arbacia lixula
Source: Int J Mol Sci. 2023 Feb 18;24(4):4136. doi: 10.3390/ijms24044136 (PMC9963837; doi:10.3390/ijms24044136)
Supplement: Supplementary file 1 [file ijms-24-04136-s001.zip › ijms-2169639-supplementary.pdf]

International Journal of
Molecular Sciences

Article

Polymer chemical identity as a key factor in microplastic-insecticide antagonistic effects during embryogenesis of sea urchin *Arbacia lixula*

Petra Burić [1], Ines Kovačić [2], Lara Jurković [3], Serkan Tez [4], Rahime Oral [4], Nediljko Landeka [5] and Daniel M. Lyons [3,*]

[1] Faculty of Natural Sciences, Juraj Dobrila University of Pula, 52100 Pula, Croatia
[2] Faculty of Educational Sciences, Juraj Dobrila University of Pula, 52100 Pula, Croatia
[3] Center for Marine Research, Ruđer Bošković Institute, 52210 Rovinj, Croatia
[4] Faculty of Fisheries, Ege University, 35100 Bornova, Turkey
[5] Teaching Institute of Public Health of the Istrian County, 52100 Pula, Croatia
* Correspondence: lyons@irb.hr

1. Supplementary Information

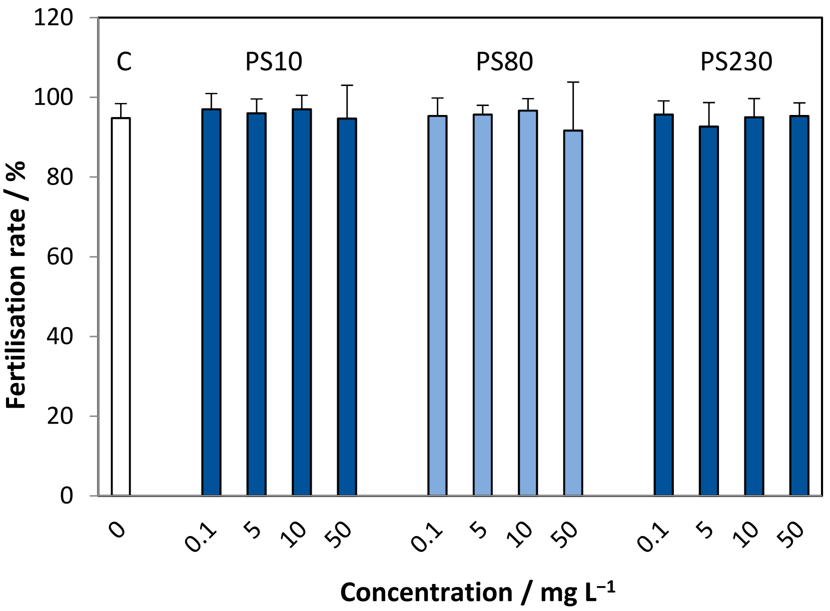

Figure S1. Fertilisation success of *A. lixula* sperm after exposure to 10, 80 and 230 μm PS microparticles of various concentrations (C – control).

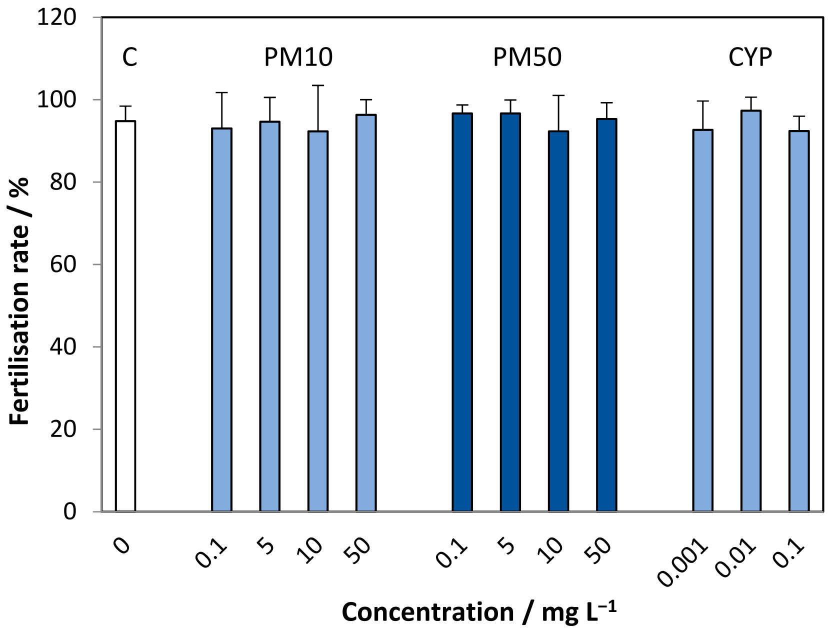

Figure S2. Fertilisation success of *A. lixula* sperm after exposure to 10 and 50 µm PMMA and cypermethrin over a range of concentrations (C – control).

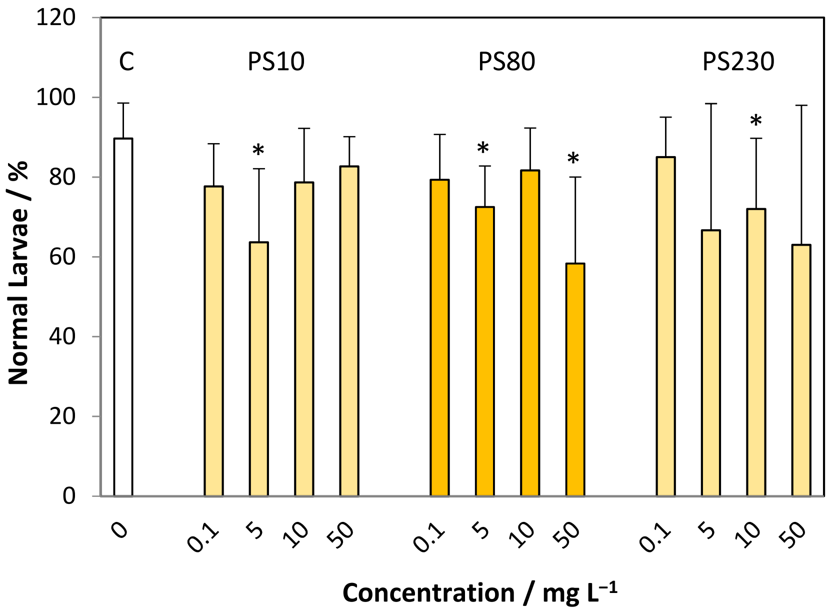

Figure S3. Normally developed offspring of sperm exposed to 10, 80 and 230 µm –diameter PS microparticles of various concentrations (C – control). Significance level, *p <0.05.

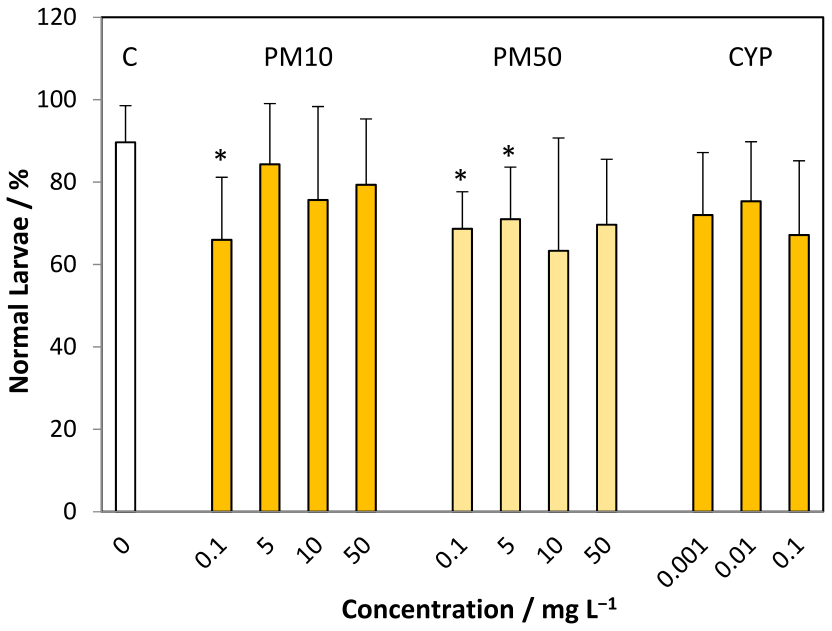

Figure S4. Normally developed offspring of sperm exposed to 10 and 50 μm –diameter PMMA and cypermethrin over a range of concentrations (C – control). Significance level, *p <0.05.

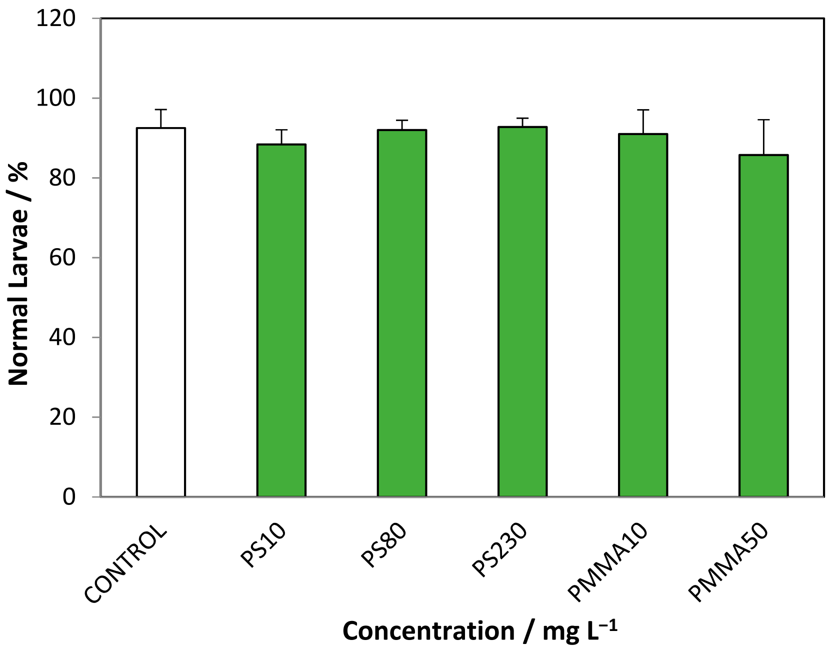

Figure S5. Normally developed offspring of sperm pre-treated with microplastic leachate (50 mg L^{-1} microplastic in filtered seawater for 1 month).
